# The Experience of Women with Obstetric Fistula following Corrective Surgery: A Qualitative Study in Benadir and Mudug Regions, Somalia

**DOI:** 10.1155/2018/5250843

**Published:** 2018-09-27

**Authors:** Adam A. Mohamed, Abiodun O. Ilesanmi, M. David Dairo

**Affiliations:** ^1^Pan African University Life and Earth Sciences Institute (PAULESI), University of Ibadan, Ibadan, Nigeria; ^2^Department of Public Health, Faculty of Health Sciences, Global Science University, Galkayo, Somalia; ^3^Department of Obstetrics and Gynecology, University College Hospital, University of Ibadan, Ibadan, Nigeria; ^4^Department of Epidemiology and Medical Statistics, College of Medicine, University of Ibadan, Ibadan, Nigeria

## Abstract

Obstetric fistula is a severe maternal morbidity which can have devastating consequences for a woman's life and is generally associated with poor obstetric services leading to prolonged obstructed labour. The predisposing factors and consequences of obstetric fistula differ from country to country and from community to community. The World Health Organization estimated that more than 2 million women in sub-Saharan Africa, Asia, the Arab region, Latin America, and the Caribbean are living with the fistula, and some 50,000 to 100,000 new cases develop annually with 30,000–90,000 new cases developing each year in Africa alone. This study aimed at describing and exploring the experiences of women living with obstetric fistulas following corrective surgery in Benadir and Mudug regions, Somalia. Women living with obstetric fistula who had surgical repairs at Daynile and GMC fistula centers and key informants were identified purposively. Twenty-one individual in-depth interviews among women with obstetric fistula and eight key informant interviews were conducted. Thematic analyses were used. Codes were identified, and those codes with similar connections were organized together as to form themes. Detailed reading and rereading of the transcribed interviews were employed in order to achieve and identify themes and categories. Themes, categories, and subcategories illustrating the experiences of women living with obstetric fistula emerged from the thematic analysis of individual in-depth and key informant interviews. These were challenges of living with OBF which include “wounds around genitalia, bad odour, incontinences of urine and feces, stigma, isolation, divorce, powerlessness, dependency, financial constraints, and loss of healthy years” and coping mechanisms which include “withdrawal from the community and improved personal hygiene.” Women with obstetric fistula experience serious health and social consequences which prevents them fulfill social, family, and personal responsibilities. We recommend expansion of BEmONC services to underserved areas, capacity building for local OBF surgeons, and improved media campaign and birth preparedness at community levels.

## 1. Introduction

Obstetric fistula is one of the most serious tragedies in childbirth injuries. It is a medical condition that involves an abnormal opening between the bladder and the vagina (vesicovaginal fistula) or between the rectum and vagina (rectovaginal fistula), or both, leading to uncontrolled leakage of urine and/or feces caused by prolonged, obstructed labour, without access to timely, high-quality medical treatment [1]. The prolonged, constant pressure of the fetal head in the birth canal cuts off the blood supply to the soft tissues surrounding the woman's bladder, rectum, and vagina.

Somalia has some of the worst maternal health indicators in the world. The fertility rate is very high as are infant and maternal mortality rates (MMR of 850 per 100,000 live births), malnutrition is chronic, early marriage is common, and most births are delivered at home without the presence of a skilled birth attendant (attended by TBAs). Somalia is also the leading country in female genital mutilation “FGM” (98%) and has the highest rate of Type III FGM (infibulation called pharaonic), with 79% of all Somali women having undergone the procedure, which is a major contributing factor for obstetric fistula [2]. Women in Somalia live in a highly insecure context where healthcare infrastructure and maternal health programs have been disrupted and limited in availability for decades. Facilities tend to be dilapidated, basic equipment and medications are in short supply, and there are few trained medical personnel in the country. All these factors are contributive to the high rate of maternal mortalities and morbidities such as obstetric fistula [3].

Obstetric fistula can be caused by prolonged labour which is mostly unrelieved, lack of access to a C-section, and low quality of C-section which can lead to iatrogenic fistula. A study found that iatrogenic fistulas occur in women who faced emergency obstetric surgery, often to address a ruptured uterus. Majority of women with iatrogenic fistulas had a history of previous cesarean section, suggesting that women who undergo C-section are at heightened risk for iatrogenic fistula during a subsequent surgery [4].

Women who experience obstetric fistula suffer constant incontinence of urine or feces or both, shame, anxiety, social segregation, and other health and social problems. It is estimated that more than 2 million young women live with untreated obstetric fistula in Asia and in sub-Saharan Africa [5, 6]. Obstetric fistula victims often experience feelings of powerlessness, physical injury, emotional breakdown, depression, divorce, erosion of social capital, and loss of health years [7]. UNFPA reported that 50,000–100,000 new cases develop annually in which the majority of the cases (99%) occur in the developing world.

Exploring and describing the experience of obstetric fistula patients and sharing with individual full stories of their own feeling will fill the gap of no baseline data, information, and stories of the occurrence, severity, consequence, and impact of the tragedy of obstetric fistula among Somali women. Discovering and documenting the plight of women with obstetric fistulas in their own voices excavates insight into the nature of this demoralizing problem and thus assists as a call for policymakers concerned with reproductive health to discourse the complex issues that propagate this preventable condition. The study aimed at exploring and describing the experiences of women who had obstetric fistulas following corrective surgery in Benadir and Mudug regions, Somalia. Specially, our research focused on the challenges that obstetric fistula victims experience and their coping mechanisms.

## 2. Materials and Methods

### 2.1. Study Area, Study Participants, and Sampling

The study was conducted in Benadir and Mudug regions, Somalia. The participants were recruited from Daynile Hospital (Benadir region) and Galkayo Medical Center (Mudug region). Benadir region or Mogadishu, the Somalia capital city, is the most populous region in Somalia which is located in the coastal area of the Indian Ocean. On the other hand, Mudug region is the most centrally located region in Somalia. The region contains two federal states namely Puntland in the north and Galmudug in the south. Although the country has some tertiary medical care facilities that are equipped to do fistula repair, there are no trained Somali doctors who can do fistula repair, and the whole country relies on foreign doctors who come for campaign [8].

Purposive, nonprobability sampling of women with obstetric fistula and key informants were employed, and the information was gathered. The research investigator with one assistant carried out 21 face to face in-depth interviews for obstetric fistula survivors who had corrective surgery in Daynile and Galkayo medical center hospitals in Benadir and Mudug regions, respectively, and 8 key informants of family members, traditional birth attendants, professionals (doctors, midwives, and nurses), policy makers, and fistula donor staffs who had first-hand knowledge about the community and provided insights into the nature of the obstetric fistula problem and gave recommendations for solutions. They were purposively selected based on the information they possess and relevance to obstetric fistula. The sample size was based on information need in which we stopped after we realized that we reached the information we needed and data saturation in which we reached data saturation on 21st respondent in the in-depth interviews who were in the hospital for recovery of their fistula operations (the participants were interviewed in the hospital within 4 weeks after surgery) and 8th respondent in the key informant interviews.

The investigator fully explained the information sheet to the participants which contained the purpose of the research, procedures, and benefits as well as the associated emotional risk of having to remember the challenges.

### 2.2. Study Design

An institution-based qualitative descriptive approach was chosen to investigate the live experiences of women with obstetric fistula after corrective surgery. This qualitative descriptive approach was done using individual in-depth interview on women with obstetric fistula and key informant interviews from participants of different sectors related to obstetric fistulas. In order to minimize recall bias since participants were asked about their live experiences, respondents were given sufficient time for adequate recall of long-time memories as to solicit full experiences. Also high-quality interview guides (interview guide related to obstetric fistula that was approved by experienced obstetric fistula consultant which was previously applied to other countries such as Africa) for both in-depth and key informant interviews were used as to minimize unwanted and irrelevant stories. And lastly, the research interviewer and the assistants had full background on the types of biases that can mislead and deflect the study. To clarify the questions, the interview guide was pretested with two obstetric fistula survivors who were recruited from Garowe city and were not included the study.

### 2.3. Data Collection Methods

#### 2.3.1. In-Depth, Open-Ended Interview

Twenty-one women who had undergone surgical repair of obstetric fistula were interviewed using an in-depth, open-ended interview guide. Before the start of the face-to-face interview, the obstetric fistula survivors were asked to complete a short personal profile questionnaire to capture out the information related to characteristics of the women (Table 1) like age, residence, marital status, literacy, number of children, occupation, obstetric history, and corrective surgery-related issues.

The information sheet, consent form, and the interview guide for obstetric fistula survivors were all translated from English to the local language “Somali language” by English-Somali translator. Data were collected with the local language “Somali” using digital audiotape recording. The investigator asked permissions from participants for tape recording before he started the interviews. The recorded audio were transcribed (converted audio data to texts), and then the transcribed texts were translated from Somali back to English for analysis. The purpose of the audio recording was to accurately record the information from the participants. Only one participant was below the age of 18 during the interview, and we found her assent and also her mother was asked to sign the consent form. All the interviewed respondents knew local name for obstetric fistula “Isku-furan” and have heard this name before.

#### 2.3.2. Key Informant Interview

Key informant interviews were held with eight people who had first-hand knowledge and involved obstetric fistula patients including family members, traditional birth attendants (TBAs) and health professionals including fistula consultant, MoH and fistula donor staffs, midwives, nurses, and policy makers. The key informant interviews were conducted to obtain in-depth information and opinion regarding the research questions. Key informants contributed validation of data provided by the obstetric fistula patients and offered expert recommendations and insights.

### 2.4. Data Analysis

A person fluent in Somali transcribed the audio from the recorded in-depth and key informant interviews supplemented by the investigators field notes. The Somali transcripts were then translated back to English language for analysis. Another person who is fluent in both English and Somali crosschecked the accuracy and completeness of the translations before data were ready for coding. The themes and categories from the in-depth interviews of obstetric fistula survivors and key informants interviews are summarized in Table 2.

All the transcripts were cautiously read sentence by sentence two times in order to become familiar with the transcripts. Codes were identified, and those codes with similar connections were organized together as to form themes. Content analyses were used. Detailed reading and rereading of the transcribed interviews were employed in order to achieve and identify themes and categories which allowed finding themes that represent broader ideas, categories, and subcategories that were more specific. This process facilitated the investigator to cross-check all the transcriptions from the respondents and recognize the data saturation point where no new information are emerging in both interviews. The narrations of the study represent respondents' descriptions of their own opinion and experiences with obstetric fistula. The themes and categories are summarized in Table 2.

## 3. Results

### 3.1. Sociodemographic Characteristics of the Participants

#### 3.1.1. In-Depth, Open-Ended Interviews

Twenty-one women with obstetric fistula were interviewed in both Benadir and Mudug Regions. Among the interviewed women in this study, 80.9% (*n*=17) were recruited from Daynile hospital, Benadir region, and 19.1% (*n*=4) from GMC hospital, Mudug Region. The ages of the study participants ranged from 15 to 55 years with both mean and modal age of 27 years. In the current ages of the respondents, 47.6% (*n*=10) were aged 15–24 years, 33.3% (*n*=7) aged 25–34, and 19% (*n*=4) aged 35 or above. According to the ages at which women developed fistula, majority of the participants (85.7% (*n*=18)) developed obstetric fistula at the age between 15 and 24 years. From the educational prospective of the study participants, majority (71.4% (*n*=15)) had no formal education, while only 28.6% (*n*=6) had formal education. Among those who had formal education, only one woman had education beyond secondary level. This means that, the majority of the data were found from women with no or little formal education. According to the residence of the interviewed women, 57.1% (*n*=12) were from rural areas before the development of obstetric fistula and 42.9% (*n*=9) were living urban areas.

The average years of women living with obstetric fistula were between 1 and 29 years although the majority 52.4% (*n*=11) were living with obstetric fistula for less than 4 years. One woman has been living with obstetric fistula for up to 29 years. She believed that, obstetric fistula is not a disease, but rather a curse from her husband because she was troublesome to her husband and his extended families. Another old woman who has been living with this condition for up to 18 years thought that, she suffered this condition because she did something bad to Allah and therefore it is punishment from Allah.

All the interviewed women were married before the development of the obstetric fistula; however, during the time of the interview, 47.6% (*n*=10) were divorced because of the condition, 42.9% (*n*=9) were still married, and 9.5% (*n*=2) were widowed. One young woman explained that her husband divorced her because of the suffering of the obstetric fistula as he could not tolerate the constant bad odour of the urine incontinences. Of the study participants, 85.7% (*n*=18) had only one obstetric fistula surgery, 9.5% (*n*=2) had experienced surgeries between 3 and 4, and only one woman had surgery above 4. According to the participant's place of last delivery, majority of them 76.2% (*n*=16) gave birth at home assisted by traditional birth attendances, whereas 23.8% (*n*=5) gave birth at health facility with the assistant of skilled birth attendants.

#### 3.1.2. Key Informant Interviews

Eight key informant interviews were conducted which include family members, traditional birth attendants (TBAs), and health professionals like fistula surgeons and consultants, fistula donor staffs, midwives, nurses, and policy makers who all had first-hand knowledge of either obstetric fistula or the community and provided their opinions and insights into the nature of the obstetric fistula problems and gave recommendations for solutions. The professional key informants have worked for periods ranging from 2 to 39 years, as shown in Table 3.

### 3.2. Challenges of Living with Obstetric Fistula

The Obstetric fistula survivors in this study reported different challenges of living with obstetric fistula which were termed as physical, psychosocial, and socioeconomic challenges (as stated in Table 2). These challenges were subgrouped into categories and subcategories.

#### 3.2.1. Physical Challenges

All of the study participants reported some sorts of physical challenges associated with obstetric fistula trauma. Majority of the respondents experienced some forms of physical problems such as irritations, bad smell, some body pains, weakness, wounds, and wetness because of the constant leakage of the urine and frictions created between thighs.

#### 3.2.2. Smell and Wounds

Majority of the participants experienced bad smell and wounds that are related to the consequences of obstetric fistula. The most serious physical consequence mentioned was the bad smell which was the result of tremendous leakage of the urine and/or feces and also constant wetness in their genital area.

*My husband left me when I got this problem. I lived without partner for 18 years. My husband said that he cannot cope with the smell and decided to leave me alone. I used to stay at home like a paralyzed person because I cannot go to the community meetings. They abuse me. When our neighbours see me, they say “the urinator” is coming, and they ask their friends “can you smell urine, what is smelling, where is it coming from.” I feel ashamed and talk to my self “self-mumbling” and say: yes they are talking about you. They are gossiping about you. Sometimes, some of them turn their lips up. Yes I hear. I never feel good. I don't have interest in people to seeing me.* (Dhubado, 39 years old, lived with obstetric fistula for 18 years)

Another participant stated that

*I frequently wear pads. I do change it every one hour. Yes when it's becoming wet and dirty that I can't sustain it, I have to change and wear a new one. It would be somehow fine for me to change but the most challenging is the embarrassing smell that is coming. I use sprayer perfume to suppress the bad odour.* (Safiyo, 17 years old, lived with obstetric fistula for 2 years)

Only one interviewer mentioned serious sores and rash that developed around her upper thigh and the area surrounding genitalia.

*This disease created sores and wounds between my thighs because I used to wear strong and heavy clothes “mostly long Hijab” to cover my whole body. I don't open my under-part and it's hot always. Yes I can't wear soft clothes. Yes I continuously wear absorbent material “pads” and hijab outfits as to minimize the tremendous wetness and odour.* (Safi, 40 years old, lived with obstetric fistula for 8 years)

#### 3.2.3. Urine and Fecal Incontinence

All the interviewed women experienced urine incontinence during the years they lived with obstetric fistula. Describing the challenges of living with obstetric fistula, all the participants were concerned about their frequent shedding and leaking of urine or feces. The women were also worried about the constant leakage of urine and/or feces which put the women to stay at home and prevented them from participating in community activities. 20 years old “Hamdi” (*pseudonym*) stated that

*I wear artificially made sanitary towel every time to limit the embarrassing leakage of urine. Yes when I acknowledge that the material is wet, I do change it. Walahi I don't stay without sanitary pads “locally known as xafayad.”* (Hamdi, 20 years old, lived with obstetric fistula for 2 years)

Another survivor explained the effects of frequent urination which is life threatening and leads to community isolation.

*I was not feeling well. Walahi if you have fistula that is the end of your life. Yes because you are not free, you can't maintain normal daily life. You can't sit normally. The urine can come anytime so you must be ready to run to the toilet. For me I don't use soaking clothes to prevent the urine incontinence except if am going outside my home.* (Amino, 19 years old, lived with obstetric fistula for 2 years).

Another participant said:

*My husband said: “I can't stay with a woman urinating every minute” and he decided to leave me and seek a new life. Yes that Sheikh “the husband” was not happy to live with me. Even me, I was feeling ashamed to face my husband. I can't carry out my daily duty*. (Aisha, 18 years old, lived with obstetric fistula for 3 years)

Five of the twenty-one interviewed women in the in-depth interview experienced incontinences of urine and feces simultaneously.

*Both my urine and feces are coming out. I always stay in the home with no one. Yes I'm alone. I am exhausted and tired of Walahi. With the help of Allah, I'm hoping to regain my normal life after this repair. This is my last hope.* (Kaafiyo, 27 years old, lived with obstetric fistula for 4 years)

The above quotes from the experiences of women living with obstetric fistula reveal that women who develop obstetric fistula suffer continues dribbling of urine and feces which prevents them from attending community gatherings such marriage and wedding ceremonies, mourning ceremonies, and community festivals. These quotes also describe, how women with obstetric fistula manage the incontinence of urine and feces. Women mentioned that, they usually wear sanitary pads or hijab outfits to minimize and limit constant dribbling of urine.

### 3.3. Psychosocial Challenges

The study participants narrated that they experienced psychological challenges due to the immediate or long-term consequences of obstetric fistula which includes stigma and isolation, reduced social support, and disrupted marital relationships.

#### 3.3.1. Stigma and Isolation

All the interviewed women complained that they suffered continuous stigma and isolation which is the effect of the urine incontinence and the odour. The degree of the stigma and discrimination was however different among survivors. For instance Muno (*pseudonym*) is 27-year-old obstetric fistula victim. She experienced dismissal from her small job (cloth washer) due to the neighbouring family's intolerance of her constant bad odour.

*I was domestic worker, I used to wash clothes of the neighbouring families. Some of the families heard that I'm a urinating woman and they chased me. Yes they said: we don't want you, you are not clean (you are dirty). I was really stigmatized by the neighbouring families. You lose your job because of this condition. The worst consequence of the OBF is the isolation.* (Muno, 27 years old, lived with obstetric fistula for 8 years)

Also the key informants narrated that women with obstetric fistula face different challenges from their community which include stigma and isolation. According to KI01 (policy maker), obstetric fistula survivors experience marginalization and stigma from their own communities:

*One of the first thing that obstetric fistula patients face is that, their husbands will abandon them, their family members will abandon them, and also the society may abandon them, so they will be put in a corner separately from the rest of the family. Yes that is why they develop serious psychological problems.* (KI01, policy maker)

Another woman who is obstetric fistula victim explained that she faced stigma from the neighbouring families; Muniro, 26-year-old woman lived obstetric fistula for nine years, narrated that she was constantly stigmatized. This surgical repair is her seventh repair since all the previous six surgical repairs were unsuccessful.

*I was sick for nine years because of this fistula condition. Every year I go to hospital and I don't have successful surgery. Yes all the people in this small village “Halabokhad” have heard about my problem so whenever I come outside, they gossip about me. Yes Walahi I can't go to community meetings in our village. My shopping is limited.* (Muniro, 26, lived with obstetric fistula for 9 years)

The interviewed key informants and women with obstetric fistula described that fistula is not only a medical condition but also interferes with the social integration. One of the key informants “KI04, nurse” explained that

*Obstetric fistula is not only a medical condition but it's also more of social and cultural problem. One of the first thing that fistula patients face is the stigma from the society so they are segregated, simply because of the condition they are living with.* (KI04, nurse)

Regarding the above narrations from both key informants and women living with obstetric fistula, the women face abuse, rejection, abandonment, and ostracization from both their couple and the community.

#### 3.3.2. Disrupted Marital Relationships and Immediate Divorces

The interviewed women indicated that they cannot have normal sex life in their marriage after sustaining obstetric fistula. Although some of the participants were still living with their spouses and kept their marriages, their relationship with their husband were limited. Only four women reported that they are the only wife of their husband and gets support from him. Majority of the obstetric fistula victims were either divorced or their husband turned away from them and married second wife.

*“Since I developed this problem, my husband limited his visiting to us because he has married another woman. Although I'm ok with it he shouldn't have abandoned us. I live with my four children and always I'm confined to the house. Yes he doesn't visit us. Yes we are not hearing his voice for up to six months.* (Safi, 40 years old, lived with obstetric fistula for 8 years)

Another interviewed woman declared that the least person from who she gets support is her husband.

*Yes, he didn't support me when my injury came. It was only my mother and siblings that supported me. He was the person I was getting the least support from before he lastly divorced me.* (Shukri, 22 years old, lived with fistula for 1 year)

Since the women with obstetric fistula cannot fulfill marital obligations, they were rejected and humiliated by their community. They also faced continuous abandonment from their spouse because the women failed to fulfill sexual obligations.

### 3.4. Socioeconomic Challenges

Ten of the interviewed women were divorced following the onset of obstetric fistula. Their husbands indicated that he was sickened by their condition and their inabilities to have normal sex life. In Somali society, though divorce is religiously allowed, it is shameful and affects the women more than men since they are dependent on their husbands throughout their lives. Most illiterate and unskilled women divorced by their husbands often return to their families. For instance, Safiyo (*Pseudonym*) is 17 years old obstetric fistula victim. She was a domestic worker in a neighbouring family. She experienced dismissal from her job due to the family's intolerance of her constant bad odour.

*I was working as a domestic worker, I used to sweep and clean the house, wash clothes, cook foods and sometimes take care of the children of that family. They heard that I cannot control urine. Yes I was frequently changing the clothes and wear pads. Eventually they sacked me. They said: “we don't want a urinating girl any more.” I lost my job.* (Safiyo, 17 years old, lived with obstetric fistula for 2 years)

#### 3.4.1. Dependency and Powerlessness

Based on the religion and cultures in the Somali society, the wives are directly dependent on their husbands. All of the interviewed women indicated that they are still struggling to survive and waiting for support from either their husbands or their extended families. Only one participant “Safi” reported that she is dependent and gets support from her children. For instance, Hamdi, 20 years old, explained that she was a farmer before she sustained this condition, but currently unable to continue farming and lives on what she gets from her family.

*I used to cultivate my farm every season like the people in this area but after developing this condition, I adopted to stay home and wait on what Allah gives me.* (Hamdi, 20 years old, dependent to her family)

Another woman reported that, she lost her small business due to the frequent pain and the leakage of the urine which made it difficult for her to continue her business. One participant emphasized the powerlessness that she experienced throughout her life. This limited power of the women in the Somali society was related to the domestic work that girls do during childhood followed by other responsibilities with in the family. Women in this society face limited freedom followed by dependency and low status of women. For example, 27-year-old Muno noted that

*In our community, women don't have power and freedom to decide. They are only here to be prepared and arranged for marriage. I didn't attend school in my life, I regret every minute Walahi. If I had the chance to go to school in my earlier years” …HAH… [Self-blaming and regretting]… I wouldn't feel the powerlessness and dependency I'm feeling now.* (Muno, 27 years old, lived with obstetric fistula for 8 years)

#### 3.4.2. Profound Poverty and Financial Constraints

All the respondents had insufficient income. In the meantime, all of them had to use more money to keep themselves clean and neat and at the same time, seek medical treatments to recover for their obstetric fistula problem. According to a family member of the obstetric fistula patient, the effect of this medical condition limited their financial income. He stated that

*She was in good health, she used to herd the family goats in the day time. Yes, as we are nomads, women are responsible for goat herding and mostly constructs the portable family house which the women ties the roofs to support the hut, but now she can't participate in any activities in the family. Yes, she needs daily support and family members to think how they can give her financial and moral support.* (KI06, family member of the victim)

One of the interviewed women experienced financial constraints having lost her livelihood and income sources and stated that:

*Since I developed this condition “OBF,” I was depending on one single source. That source is what I get from my family especially my parents. When I want to go and pay something, I have to beg my parents and wait for some days. Everything in this world is money. The health care is too costly here in Somalia.* (Barni, 21 years old, lived with obstetric fistula for 4 years).

### 3.5. Coping Mechanisms

The interviewed women in this study revealed different efforts to normalize and cope with their emotions which resulted from obstetric fistula through isolating themselves, hiding their story, and always keeping good physical hygiene and purity which provided relief and helped them calm down their stress and anxiety. For instance, 23-years-old “Maryamo” who lived with obstetric fistula for 2 years stated that

*It is not easy to live with this problem [obstetric fistula]. My concern was people not to know that I cannot control urine. I don't want them to know my disease. I frequently wash my clothes especially the sanitary pads and clean my body to prevent the odour of the urine. I was trying to keep my cleanness.* (Maryamo, 23 years old, who lived with obstetric fistula for 2 years)

Another woman explained how she copes after living with obstetric fistula for nine years and noted that

*You cannot go to the public places during community gatherings with this problem [obstetric fistula]. The people will know that you continuously urinate. The community around here will find you if you meet them. You feel ashamed and humiliated when people stare at you. I have to hide myself and stay at home. I always isolate myself because people in this community see me as if my body is rotten.* (Muniro, 27 years old, who lived with obstetric fistula for 9 years)

Another participant explained that

*I frequently change my clothes and mostly use heavy robes “long hijab outfits” to avoid the embarrassing odour. When I'm going outside, I wear three garments “Diracyo (local name)” with sanitary towel to reduce and control the dribbling of the urine.* (Habibo, 20 years old, who lived with obstetric fistula for 3 years)

Withdrawal from the community and improved personal hygiene were the two strategies that women with obstetric fistula used to cope with their condition. As the above statements describe, women who are living with obstetric fistula were frequently changing their clothes to avoid the bad odour and wetness resulted from obstetric fistula.

## 4. Discussion

The challenges encountered by the interviewed women were similar to the reports by previous researches [7, 9–11] that obstetric fistula brings consequences of physical challenges, including sores, skin rashes, and wounds around the genitalia, bad smell, and incontinences of urine and feces. Psychosocial challenges include stigma and isolation, disrupted marital relationships, divorce, suffering and loss of the baby, and socioeconomic challenges, which includes powerlessness, dependency, limited social support, financial constraints, profound poverty, and loss of healthy years. The findings of this study is also consistent with those studies conducted in Ethiopia [6, 12, 13], Burundi [14], DR Congo and Bangladesh [15], Kenya [9], Ghana [16], Nigeria [17], and Tanzania [11] regarding the physical, psychosocial, and socioeconomic challenges experienced by obstetric fistula survivors.

The study found that obstetric fistula patients have fears to retain and share sexual activities with their husbands in the future. This finding is consistent with a study conducted in West Pokot, Kenya [9] which concluded that fistula victims still have fear to have sex with their husbands even after successful repairs.

In this study, women affected by obstetric fistula usually encounter immediate divorce as shown by the study result which was about 47.6% (*n*=10). Divorce is common in obstetric fistula patient. Due to the onset of obstetric fistula, some husbands divorce their wives because of bad odour and leakage of the urine that is disgusting to them. Others divorce their wives because of fistula survivors cannot satisfy them sexually while others divorce because the obstetric fistula women cannot produce another children in the future and it may take them years to recover. This was similar to previous studies conducted by Landry et al. [18] and Gebresilase [7]. In this study, about two-fifths are still married although their husbands decided to marry second time. This is true and corroborates the explanations of the key informants that, due to the condition of their wives, the husbands always try to find second or third wives.

Another research conducted in Malawi [19] reported that obstetric fistula victims do not get much support from their husbands because of the polygamous practices. Our study finding was also similar in its report that some of the husbands neglect their wives because of the fistula condition and prefer to stay with their second or third wives.

This study has similar outcome with several studies conducted in African countries [9, 15, 16, 20], which agreed that women who lived with obstetric fistula frequently wash their clothes and use perfumes to cover the offensive odour. Studies conducted in Kenya and Nigeria [9, 17] reported that husbands ostracize their wives after developing obstetric fistula and do not want to share life and eat with them at both house and family events. This study reported similar findings on the victim's inability to satisfy their husbands sexually and the neglect they suffer because of the disease.

Previous reports by Kimani et al. [10] and fistula foundation [3] revealed that some women with obstetric fistula suffer from “foot drop,” which is the inability to walk properly without help, due to injury to the common fibular and sciatic nerves caused by prolonged labour. Similarly, key informants in our study revealed that women who develop obstetric fistula suffer nerve injuries that can cause foot drop and long-term walking impairment. The key informants acknowledged that women with obstetric fistula can develop foot drop; but, among the interviewed women who lived with obstetric fistula, none of them reported that they experienced nerve injury or foot drop.

It was further revealed that women living with obstetric fistula face daily life challenges including inability to attend community gatherings, have sexual relationship with their husbands, earn money, attend religious prayers, work and participate in small businesses, and eat with others [18]. Our study also corroborates this finding. Majority of the participants explained that they were not attending religious prayers since they developed obstetric fistula. The participants also lamented lack of participation in social activities; they do not even have enough courage to attend community gatherings like festivals or wedding ceremonies because of the wound and bad smell resulted from the obstetric fistula. Concerning personal earnings, interviewed women reported that they are facing financial constraints and their income is almost zero now.

Despite the fact that majority of the women interviewed suffered neglect and abandonment from their husbands and communities, family and relatives were the most reliable source of income and moral support for women living with obstetric fistula. Although there was a limited resource, family members including parents, siblings, and their children play a key role in supporting and rendering assistance for food, water, clothes, and personal hygiene. The participants revealed that they were getting assistance from their family members when seeking medical treatment.

Respondents in this study exhibit similar efforts to normalize and cope with their emotions through isolating themselves, hiding their story, and always keeping good physical hygiene and purity which provided relief and helped them calm down their stress and anxiety. This study is interested in the various mechanisms and strategies that obstetric fistula survivors use to cope with their condition. As reported by previous studies [7, 16, 17, 21], people with medical illnesses and conditions use different coping strategies as to manage their life problems. Similarly, the study participants explained various techniques and strategies they use to cope with their condition. The participants isolated themselves from other people, put on Hijab outfits to cover the bad smell, and always carry extra clothes and water in order to control the urine incontinence and bad odour.

As reported in previous studies [7, 16, 20], participants in this study also use artificially made sanitary towel in order to reduce or control the embarrassing odour and to absorb continuous leakage of urine. They also frequently change clothes and use Hijab outfits to cope with the problem. In order to control and prevent wetting seats and chairs because of the incontinences of urine and feces, the victims dressed in three or four “diracyo”, the traditional wears Somali women wear.

The interviewed women use withdrawal strategy to cope with their problems. They always avoid sitting and staying with or attending community gatherings. Many of them stay at home every time and restrict their movements because of the fear of stigma from the community and the uncontrollable leakage of urine and feces.

## 5. Conclusion and Recommendations

The direct statements of women living with obstetric fistula from two regions of Somalia (Benadir and Mudug) and key informants involved in obstetric fistula presented in this study ensure the validation of the study findings. This study finding addresses the experiences of women living with obstetric fistula and their coping mechanisms in Benadir and Mudug regions, Somalia. The experiences they lived with were almost similar to other findings in sub-Saharan African countries, especially East African societies which have related socioeconomic issues regarding the obstetric fistula challenges. The study findings demonstrate the challenges that women with obstetric fistula experience. These include physical challenges “wounds around the genitalia, bad smell, and incontinences of urine and feces,” psychosocial challenges “stigma and isolation, disrupted marital relationships, divorce, suffering, and loss of the baby,” and socioeconomic challenges “powerlessness, dependency, limited social support, financial constraints, profound poverty, and loss of healthy years.”

Findings of this study highlighted how the poverty, illiteracy, women's dependency to their husbands, low status of women, traditional beliefs and practices, and limited antenatal care utilization twist together to produce the devastating problem of obstetric fistula in Somalia. Women living with obstetric fistula cannot fulfill social, family, and personal responsibilities due to the obstetric fistula challenges. The basic health care system in Somalia is very weak and insufficient to meet essential obstetric care of pregnant women.

### 5.1. Recommendations

1. The basic health care system in Somalia is very weak and insufficient to meet essential obstetric care of pregnant women. Basic Emergency Obstetric and Newborn Care “BEmONC” should be expanded to all areas to address prolonged and obstructed labour which in turn prevents women to develop obstetric fistula.
2. Since the country mostly relies on foreign fistula surgeons, training and capacity building of local fistula surgeons should be provided.
3. Fistula facilities should be upgraded to perform fistula repair surgery.
4. Continuous awareness and extensive public media campaign towards ANC attendance and birth preparedness should be implemented at community levels “generate media attention.”
5. Advocates should be designed that are working at all levels whether national, district, or community levels.
6. Community development should focus on promoting safe motherhood practices including provision of family planning, adequate child spacing of pregnancies, and focused antenatal care.
7. Increased human resource and funding for obstetric fistula including training TBAs, increasing the number of skilled birth attendants, and ensuring appropriate distribution of all the trained skilled birth attendants to all geographical locations.
8. Government should bring means of social reintegration and women empowerment mechanisms after surgical correction of women with obstetric fistula.

## Figures and Tables

**Table 1:** Participants' background profile.

Variable	Characteristics		
	Category	Number	Percentage (%)
Current age (years)	15–24	10	47.6
	25–34	7	33.3
	35–44	2	9.5
	45–54	1	4.8
	55 and above	1	4.8

Age at OBF occurrence (years)	15–24	18	85.7
	25–34	2	9.5
	35–44	1	4.8
	45–54	0	0.0
	55 and above	0	0.0

Region	Benadir	17	81.0
	Mudug	4	19.0

Residence	Rural	12	57.1
	Urban	9	42.9

Education	Formal education	6	28.6
	No formal education	15	71.4

Occupation	Unemployed	15	71.4
	Employed	6	28.6

Current marital status	Married	9	42.9
	Widowed	2	9.5
	Divorced	10	47.6

Place of last delivery	Home	16	76.2
	Health facility	5	23.8

Number of years lived with obstetric fistula	1–3	11	52.4
	4–6	4	19.0
	7–9	3	14.3
	Above 9	3	14.3

Number of surgeries	1–2	18	85.7
	3–4	2	9.5
	Above 4	1	4.8

Parity	Primigravid	9	42.9
	Para 2–4	7	33.3
	Para 5 or more	5	23.8

Source: field data 2017.

**Table 2:** Themes and categories that emerged.

Themes	Categories
Challenges of living with obstetric fistula	(i) Physical challenges
	(a) Mell and wounds
	(b) Urine and fecal incontinence
	(ii) Psychosocial challenges
	(a) Stigma and isolation
	(b) Disrupted marital relationships and immediate divorces
	(iii) Socioeconomic challenges
	(a) Dependency and powerlessness
	(b) Profound poverty and financial constraints

Coping strategy	(iv) Withdrawal from the community
	(v) Improved personal hygiene

Source: thematic analysis of the field data 2017.

**Table 3:** Characteristics of key informants.

KI code	Sex	Age (years)	Position	Occupation	Years of experience	Residence region
KI01	M	74	Policy maker	RH consultant	36	Benadir
KI02	M	73	Consultant	Fistula surgeon	39	Mudug
KI03	M	31	General physician	UNFPA staff	5	Benadir
KI04	F	39	Nurse	Qualified nurse	10	Mudug
KI05	M	30	General physician	Fistula assistant	2	Mudug
KI06	M	71	Family head	Jobless	—	Benadir
KI07	F	67	Midwife	Midwifery	38	Mudug
KI08	F	64	TBA	TBA	32	Benadir

KI: key informant; RH: reproductive health; TBA: traditional birth attendants. Source: field data 2017.

## Data Availability

The data used to support the findings of this study are available from the corresponding author upon request.
